# Bisphenol A and Its Analogue Bisphenol S Inhibit Cholinergic Neurotransmission at the Tripartite Colonic Myenteric Synapse of CD1 Mice by Targeting Interstitial Cells of Cajal

**DOI:** 10.3390/ijms26178279

**Published:** 2025-08-26

**Authors:** Krystyna Makowska, Cátia Vieira, Isabel Silva, Yoce Aprianto, Diogo Silva, Catarina Bessa-Andrês, Ana Lopes, Sławomir Gonkowski, Paulo Correia-de-Sá

**Affiliations:** 1 Department of Clinical Diagnostics, Faculty of Veterinary Medicine, University of Warmia and Mazury in Olsztyn, Oczapowskiego 14, 10-957 Olsztyn, Poland; 2 Laboratório de Farmacologia e Neurobiologia, Center for Drug Discovery and Innovative Medicines (MedInUP) and RISE-Health: Health Research Network, Instituto de Ciências Biomédicas de Abel Salazar, Universidade do Porto (ICBAS-UP), 4050-313 Porto, Portugal; cavieira@icbas.up.pt (C.V.); issilva@icbas.up.pt (I.S.); yoce.aprianto@inrae.fr (Y.A.); diogo.a.r.silva@hotmail.com (D.S.); up201703814@edu.fc.up.pt (A.L.); farmacol@icbas.up.pt (P.C.-d.-S.); 3 Department of Clinical Physiology, Faculty of Veterinary Medicine, University of Warmia and Mazury in Olsztyn, Oczapowskiego 13, 10-957 Olsztyn, Poland

**Keywords:** bisphenol, mice, enteric nervous system, colon, myenteric synapse

## Abstract

Bisphenol A (BPA) and bisphenol S (BPS) are frequently used in the plastic industry. Despite significant alimentary exposure, their effects on the gastrointestinal (GI) tract remain largely unknown. Cholinergic and/or purinergic neurotransmission facilitates GI tract motility and secretion, indirectly controlling the absorption and toxicity of xenobiotics. Hence, this study examined the neurochemical effects of BPA and BPS in the tripartite cholinergic myenteric synapse of CD1 mice colon. Short time exposure to both bisphenols showed a partial loss of VAChT-positive neurons and Ano-1-positive interstitial cells of Cajal (ICCs), without affecting the amount of glial cells labelled with S100β. Both bisphenols reduced the spontaneous myographic activity and the release of [^3^H]acetylcholine ([^3^H]ACh) and adenosine from stimulated myenteric neurons and pacemaker ICCs, respectively, without affecting the outflow of ATP. Overall data suggest that both bisphenols inhibit the cholinergic neurotransmission of CD1 mice colon by affecting the amount and/or function of ICCs at the tripartite myenteric synapse.

## 1. Introduction

Bisphenols are widely used in the chemical industry for the production of polycarbonate plastics and epoxy resins and, therefore, are present in a wide variety of consumer products, such as food and beverage cans, thermal papers, medical equipment, and baby bottles, as well as toys for children [1,2]. Until recently, bisphenol A (BPA; also known as 4,4′-dihydroxy-2,2-diphenylpropane) was one of the main components in the production of plastic ware [3,4]. However, countless studies demonstrate that BPA-containing products used on a daily basis have a detrimental impact on health due to their chemical resemblance to oestrogens [5]. Progressive replacement of BPA in the plastic industry resulted in increments of the application of bisphenol S (BPS; 4,4’-sulfonyldiphenol or bis(4-hydroxyphenyl)sulfone) by many countries [6] as a putative safer alternative in BPA-free products [7,8,9]. Although BPS was considered completely safe for living organisms, recent studies showed that BPS also exhibits oestrogen-potentiating effects, which negatively affect a wide range of physiological processes [10,11,12]. There is, however, a lack of information regarding many aspects of BPS actions, which certainly deserve further investigation.

In most living organisms, exposure to BPA and BPS toxicity occurs mainly via their absorption by the gastrointestinal tract and, therefore, the negative impact of bisphenols on health conditions may depend on their effects on gastrointestinal tract functions, i.e., epithelial absorption, exocrine secretion and peristalsis. BPA can reduce the intestinal motility [13] and the composition of gut microbiota [14], but the time-course of these changes is not fully elucidated. Moreover, BPA can damage the intestinal barrier, increasing intestinal permeability, as well as affecting the transmission of sensory inputs from the digestive tract [15]. BPA promotes oxidative stress responses, mitochondrial dysfunction and inflammation in the intestine. Previous studies by the current authors demonstrated that bisphenols alter the neurochemical coding of enteric neurons [16,17,18,19,20,21], but the mechanism(s) and the functional repercussions of these changes are yet to be determined.

The current study focused on the role of BPA and BPS on the cholinergic neuromuscular transmission in the colon of mice. The enteric nervous system (ENS) comprises two major ganglionic plexuses: the submucous plexus (SmP) located in the lamina propria of the mucous layer and the myenteric plexus (MP) located between the circular and longitudinal muscle layers [22,23,24]. While the submucous plexus controls the local gut secretion and absorption, the myenteric plexus regulates the gastrointestinal motility, as well as the secretion of enzymes from adjacent organs [22,23,24]. Besides enteric neurons playing roles in the regulation of the digestive tract functions, their interplay with non-neuronal cell populations, including enteric glial cells and interstitial cells (interstitial cells of Cajal—ICCs and fibroblast-like cells—FLCs), also contributes to gastrointestinal homeostasis and activity. In fact, the enteric neuromuscular junction is a tripartite synapse, comprising the nerve terminal, the smooth muscle fibre and a non-neuronal cell third party [25,26].

Although more than fifty neuroactive substances have been described in the ENS [27], acetylcholine (ACh) is recognised as the prime excitatory neurotransmitter throughout the digestive tract [28]. Pathological and toxicological insults dramatically affect the neurochemical coding and the amount of neuroactive substances in the ENS [29,30], most often including the main cholinergic neurotransmitter, ACh, and its co-transmitters, like ATP [27]. More recently, data highlighted changes in the structure and activity of adjacent non-neuronal cells participating in the tripartite enteric synapse. Mounting evidence indicates that the release of ATP and its metabolite, adenosine, from non-neuronal cells is critical to control gastrointestinal functions both under normal and pathological conditions [25,26]. For instance, enteric inflammation operates a shift on purinergic neuromodulation of cholinergic neurotransmission, reflecting the upregulation of ATP-releasing enteric glial cells and the depletion of ICCs, accounting for decreased adenosine overflow via equilibrative nucleoside transporters [26]. Data showed that the extracellular ATP accumulation contributes to maintaining the cholinergic tone at a minimum via the activation of P2X2 and/or P2X2/3 receptors located on cholinergic nerve fibres. This mechanism recapitulates the ancestral mechanism by which ATP released from glial cells directly (via ionotropic P2X receptors) stimulates the release of ACh from cholinergic nerves even without neuronal firing.

Thus, considering the significant role of cholinergic/purinergic neurotransmission in directly controlling digestive tract motility and secretion and indirectly regulating the absorption of toxic compounds, the current study investigated the neurochemical effects of BPA and its substituent, BPS, in the tripartite colonic myenteric synapse of CD1 mice.

## 2. Results

### 2.1. BPA and BPS Decrease the Release of [^3^H]ACh from Stimulated Myenteric Neurons Without Affecting Smooth Muscle Performance in Isolated LM-MP Preparations of CD1 Mice Mid Colon

**Figure 1:**
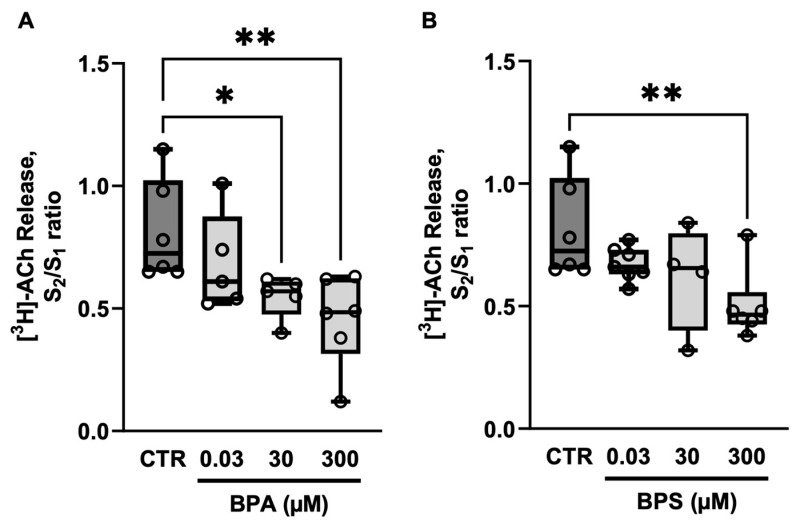
Effects of bisphenol A (BPA) (**A**) and bisphenol S (BPS) (**B**) on [^3^H]ACh release from electrically stimulated longitudinal muscle-myenteric plexus of the mouse mid colon. [^3^H]ACh release was elicited twice (S_1_ and S_2_) by EFS (200 pulses delivered at 5 Hz frequency). BPA and BPS (0.03–300 µm) were added to the superfusion fluid 8 min before S_2_ and remained until the end of the experiments. The ordinates are S_2_/S_1_ ratios obtained in the absence (CTR) and in the presence of BPA (**A**) or BPS (**B**). Boxes and whiskers represent pooled data from an *n* number of experiments (small dots). * *p* < 0.05 and ** *p* < 0.01 (one-way ANOVA, uncorrected Fisher’s LSD) represent significant differences compared to the control condition.

BPA and BPS (0.03–300 µm) concentration-dependently reduced the release of [^3^H]ACh from electrically stimulated LM-MP preparations of CD1 mice mid colon (Figure 1). The inhibitory effect of BPA was observed at concentrations above 30 µm, whereas BPS required a 10 times higher concentration (300 µm) to elicit a similar effect.

**Figure 2:**
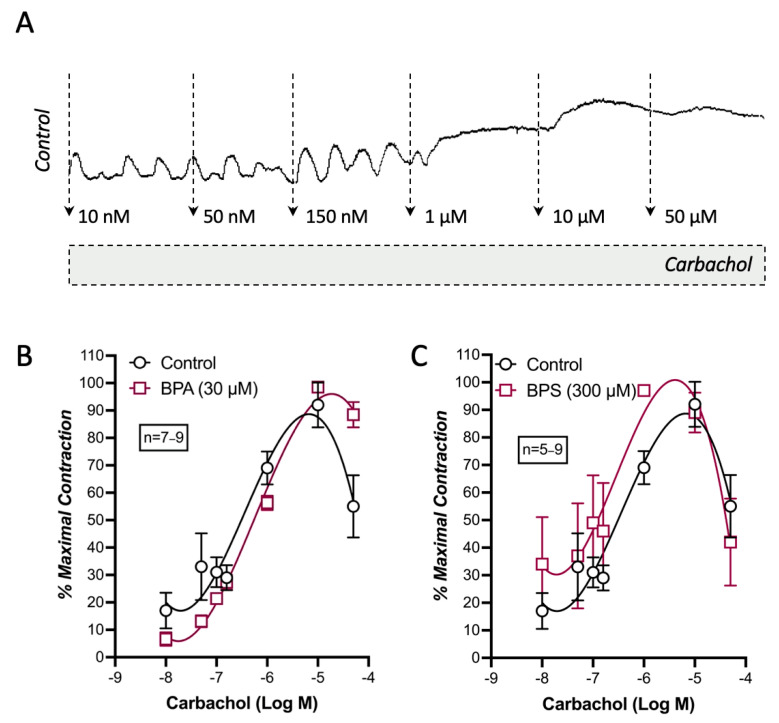
BPA and BPS display no effect on carbachol-induced myographic recordings of longitudinal muscle-myenteric plexus preparations of CD1 mice mid colon. Shown are concentration–response curves of carbachol (0.01–50 μm; **A**) obtained in the absence (Control) and in the presence of BPA (30 µm; **B**) or BPS (300 µm; **C**); these drugs were added to the incubation fluid 15 min before application of carbachol. The ordinates are the percentage of maximal contractions. The vertical bars represent ± SEM from an *n* number of experiments.

When used at equipotent threshold concentrations, BPA (30 µm) and BPS (300 µm) failed to modify carbachol-induced contractions of LM-MP preparations of CD1 mice mid colon (Figure 2).

These results suggest that both BPA and BPS cause inhibition of colonic neuromuscular transmission predominantly by reducing the release of ACh from stimulated myenteric cholinergic neurons rather than by affecting the smooth muscle contraction caused by the cholinergic agonist, carbachol.

### 2.2. Oral Administration of BPA and BPS Causes a Partial Loss of Myenteric Cholinergic Neurons and Interstitial Cells of Cajal (ICCs), Without Affecting Enteric Glial Cells Immunoreactivity in CD1 Mice Colon

The inhibitory role of BPA and PBS on electrically evoked transmitter release in LM-MP preparations of CD1 mice mid colon prompted the authors to investigate changes in the density and/or distribution of VAChT-positive cholinergic neurons two weeks after supplementation of the animals’ drinking water with BPA and BPS (50 mg/kg b.w. per day). The chemical structure of bisphenols allows them to bind to oestrogen receptors that are present in the enteric neurons, but also in non-neuronal enteric glial cells and ICCs, cooperating to control the neuromuscular transmission at the tripartite myenteric synapse [5,26]. For this reason, triple labelling experiments were performed using fluorescently tagged antibodies against specific protein markers of glial cells and ICCs, such as S100β and Ano-1, respectively.

**Figure 3:**
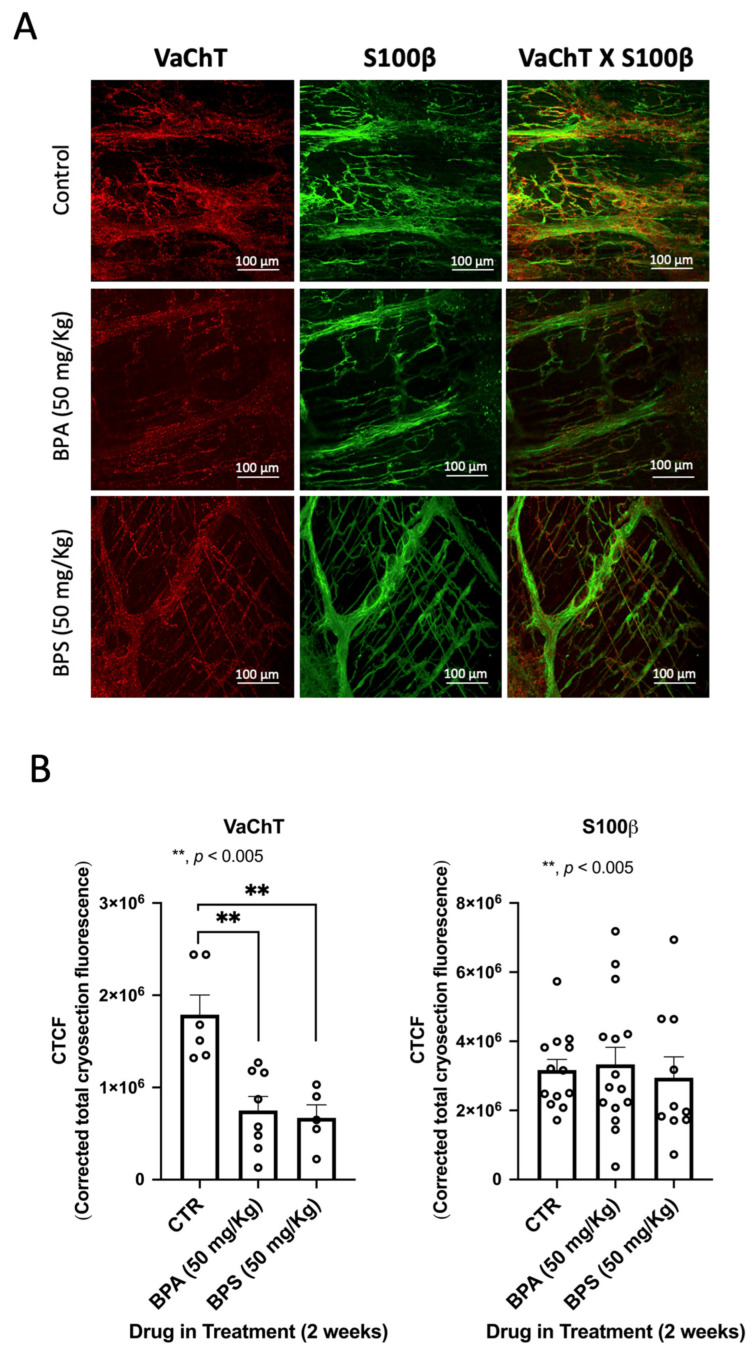
(**A**) shows confocal micrographs of whole-mount preparations of the longitudinal muscle-myenteric plexus of the colon of untreated (control) and of BPA- or BPS-treated CD1 mice stained against the vesicular ACh transporter (VAChT) and the enteric glial marker, S100β. Scale bar = 100 μm. In (**B**), the bar charts represent mean ± SEM of corrected total cryosection fluorescence (CTCF) staining against VAChT and S100β, respectively. ** *p* < 0.01 (one-way ANOVA, uncorrected Fisher’s LSD) represents significant differences compared to untreated (control) animals.

**Figure 4:**
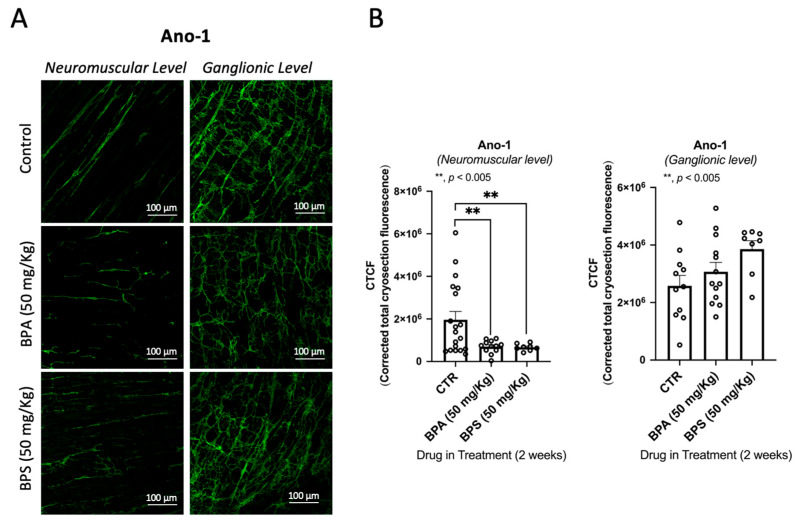
Confocal micrographs of whole-mount preparations of the longitudinal muscle-myenteric plexus of the mid colon of CD1 from untreated (control) and BPA- or BPS-treated mice stained against the Anoctamine-1 (Ano-1) taken at the myenteric ganglion level and at the longitudinal smooth muscle layer (**A**). Bar charts represent mean ± SEM of corrected total cryosection fluorescence (CTCF) staining against Ano-1, at the neuromuscular layer and at the ganglionic layer (**B**). ** *p* < 0.01 (one-way ANOVA, uncorrected Fisher’s LSD) represents significant differences compare.

We observed no clinical abnormalities in the animals receiving BPA or BPS throughout the experiments. We also did not observe differences in both the behaviour and amount of food consumed. However, confocal microscopy observations of the LM-MP of the colon of BPA/BPS-treated mice show significant (*p* < 0.05) decreases in the density of VAChT-positive cholinergic neurons, without changing (*p* > 0.05) the amount of S100β-positive enteric glial cells (Figure 3). Interestingly, treatment of CD1 mice with BPA or BPS (50 mg/kg b.w. per day) in the drinking water also depleted Ano-1-positive ICCs in LM-MP preparations isolated from the colon of these mice (Figure 4). BPA/BPS-induced ICCs depletion in the colon of CD1 mice was more evident at the neuromuscular layer (intramuscular spindle-shaped cells) than at the ganglionic level (myenteric stellate cells). Neuronal and non-neuronal cell density changes cannot be attributed to alterations in the feeding behaviour, body weight or histopathology of the colonic wall, because no significant (*p* > 0.05) differences were found between BPA/BPS-treated and non-treated groups [20].

### 2.3. Selective Blockage of ICCs Activity Prevents the Inhibitory Effect of BPA and BPS on [^3^H]ACh Release from Stimulated Myenteric Neurons of CD1 Mice Mid Colon

**Figure 5:**
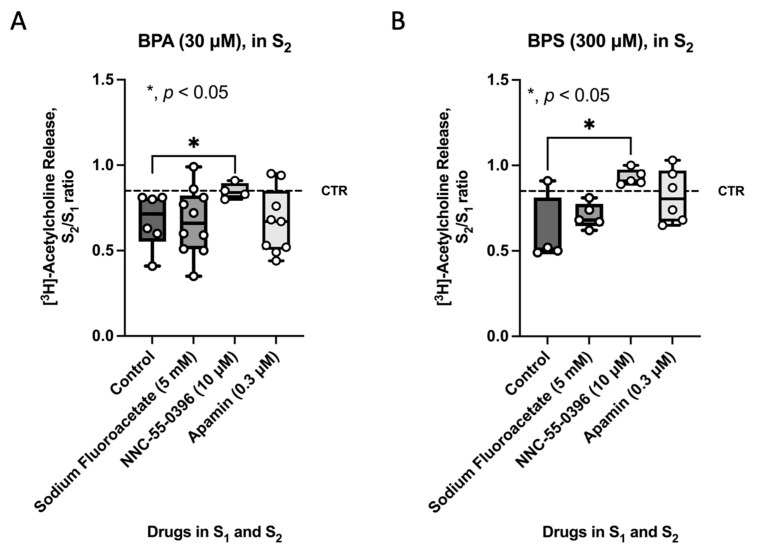
Effect of BPA (30 µm) (**A**) and BPS (300 µm) (**B**) in the presence of fluoroacetate (5 mm, a glial cell metabolic uncoupler), NNC-55-0396 (10 µm, an inhibitor of Ca_v_3 (T-type) channels in ICCs) and apamin (0.3 µm, an inhibitor of small-conductance K_Ca_2 (SK3) channels in FLCs) on nerve-evoked [^3^H]ACh release from LM-MP preparations of CD1 mice mid colon. [^3^H]ACh release was evoked twice (S_1_ and S_2_) by EFS (5 Hz frequency, 200 pulses). Fluoroacetate (5 mm), NNC-55-0396 (10 µm) and apamin (0.3 µm) were added to the incubation solution at the beginning of the release period (zero time) and remained throughout the assay, including S_1_ and S_2_; BPA and BPS were added 8 min before S_2_. The ordinates are S_2_/S_1_ ratios for the effect of BPA (30 µm) (**A**) and BPS (300 µm) (**B**) either in the absence (control) or presence of fluoroacetate (5 mm), NNC-55-0396 (10 µm) and apamin (0.3 µm). Dashed horizontal lines represent the S_2_/S_1_ ratio in the absence of any drug. Boxes and whiskers represent pooled data from an *n* number of experiments (small dots). * *p* < 0.05 (one-way ANOVA, uncorrected Fisher’s LSD) represent significant differences compared to the effect of BPA (30 µm) (**A**) or BPS (300 µm) (**B**) under the control condition.

Given the loss of Ano-1-positive intramuscular spindle-shaped ICCs in the colon of CD1 mice treated with BPA or BPS (50 mg/kg b.w. per day), the authors investigated their role in BPA-and BPS-induced inhibition of evoked [^3^H]ACh release from myenteric neurons by blocking the activity of ICCs with the voltage-sensitive Ca_v_3 (T-type) blocker, NNC-55-936 (10 μm). For comparison purposes, tests were conducted on the modulating effects of the glial cell metabolic uncoupler, sodium fluoroacetate (5 mm), and of apamin (0.3 μm), which prevents the activity of fibroblast-like cells (FLCs) by blocking small-conductance K_Ca_2 (SK3) channels. Figure 5 shows that only NNC-55-936 (10 μm), but not sodium fluoroacetate (5 mm) or apamin (0.3 μm), could prevent the inhibitory effect of BPA (30 µm) and BPS (300 µm) on nerve-evoked [^3^H]ACh from LM-MP preparations of CD1 mice colon. Data suggest that inhibition of transmitter release is highly dependent on the presence of ICCs and their functional interplay with cholinergic myenteric neurons, whereas the crosstalk with enteric glia and FLCs appears to be irrelevant.

### 2.4. BPA and BPS Decrease the Spontaneous Myographic Activity of CD1 Mice Mid Colon in a Similar Manner to That Caused by Blockage of Voltage-Sensitive Cav3 (T-Type) Channels in ICCs with NNC-55-936

**Figure 6:**
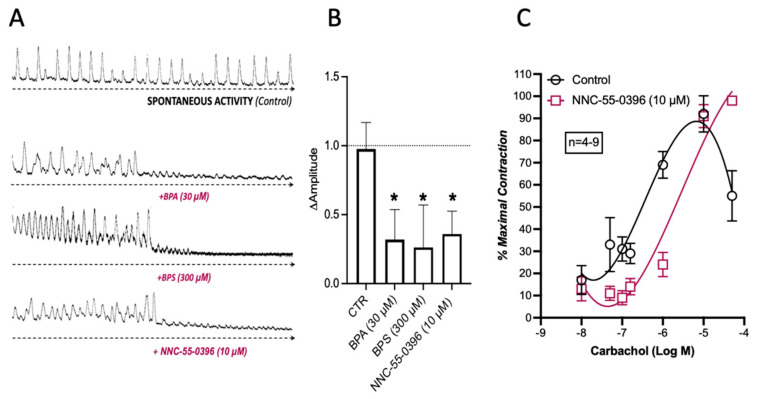
Spontaneous contractile activity of LM-MP preparations of the mice mid colon in response to BPA (30 µm), BPS (300 μm) and NNC-55-0396 (10 µm, (**A**)). (**B**) shows a histogram representative of the quantification of variation in magnitude of spontaneous contractions of LM-MP preparations in the absence and in the presence of BPA (30 μm), BPS (300 μm) and NNC-55-0396 (10 μm). (**C**) shows the concentration–response curves of carbachol (0.01–50 μm) in the absence and in the presence of NNC-55-0396 (10 µm); this compound was added to the incubation fluid 15 min prior to the application of carbachol. The ordinates are the percentage of maximal contraction. The vertical bars represent ± SEM from a number of experiments. The data are means ± SEM * *p* < 0.05 (one-way ANOVA, uncorrected Fisher’s LSD).

ICC pacemaker cells control and coordinate peristalsis in the GI tract. Therefore, the loss of ICCs and/or blockage of their activity may affect GI automatism. Here, the “in vitro” effect of BPA (30 µm) and BPS (300 µm) on the spontaneous contractile activity of colonic LM-MP preparations was compared with that observed upon blocking small-conductance K_Ca_2 (SK3) channels in ICCs with NNC-55-936 (10 μm). Figure 6 shows that BPA (30 µm) and BPS (300 µm) mimicked the inhibitory effect of NNC-55-936 (10 μm) on the amplitude, but not on the frequency, of spontaneous colonic contractions without affecting carbachol-induced contractions (see Figure 2).

### 2.5. BPA and BPS Reduce the Release of Adenosine, but Not of ATP, from Stimulated LM-MP Preparations of the CD1 Mice Mid Colon

**Figure 7:**
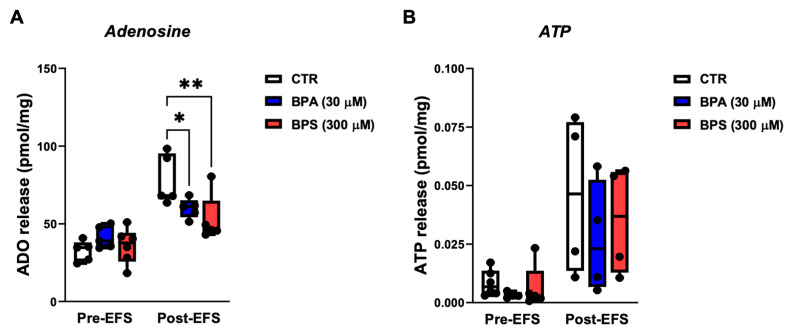
A: Effects of BPA and BPS on adenosine (**A**) and ATP (**B**) release from LM-MP preparations of CD1 mice mid colon under basal conditions (Pre-EFS) and after electrical field stimulation (Post-EFS; 3000 pulses delivered at 5 Hz frequency). BPA (30 µm) and BPS (300 µm) were added to the incubation fluid 15 min before EFS. Boxes and whiskers represent pooled data from an *n* number of experiments (small dots). * *p* < 0.05 and ** *p* < 0.01 (one-way ANOVA, uncorrected Fisher’s LSD) represent significant differences compared to the control (CTR) condition in the absence of any drug.

A previous study showed that ATP is a putative excitatory gliotransmitter at the tripartite myenteric cholinergic synapse and that the release of ATP may be uncoupled from extracellular adenosine appearance, providing the nucleoside is released from activated ICCs via the equilibrative nucleoside transporter, ENT1 [26]. Considering that BPA and BPS caused the loss and/or decreased the activity of ICCs without affecting the density and activity of enteric glia, BPA/BPS-induced changes were determined in the amount of the two important purine controllers of the GI tract function. Figure 7 shows that both BPA (30 µm) and BPS (300 µm) decreased the release of adenosine from stimulated LM-MP preparations, without significantly (*p* > 0.05) affecting the outflow of ATP. Data also support the view that both BPA and BPS primarily affect the “in vitro” activity of ICCs under these experimental conditions.

## 3. Discussion

Nowadays, plastic products are commonly used in daily human activities all over the world. Among others, polycarbonate and epoxy resins are employed for the production of bottles, food containers, medical and dental products, toys, thermal papers, furniture and furnishings, and automobile components. Until recently, BPA was a main component in the plastic industry [3,4]. However, due to countless scientific studies showing that it could have a negative impact on living organisms [5], an ever-increasing number of countries have adopted measures to restrain the use of BPA in the plastic industry [31]. It is now recognised that BPA affects the function of various organic systems, like the digestive tract, the central nervous system, the immunological system, reproductive organs and hormonal homeostasis [7,8,9]. Moreover, it has been reported that BPA can accumulate in several human tissues and organs [32], and chronic exposure to this substance can lead to some long-term dysfunctions or even organ failure and cancer development [3,4].

To overcome this constraint, other compounds have progressively replaced the use of BPA. “BPA-free” products were considered safer for human and animal use until very recently. However, like BPA, its alternatives may be leached from plastic products into water and food, absorbed into body fluids and tissues, and, therefore, may also have negative impacts on living organisms [10,11,12]. The most widely used alternative to BPA is BPS [11,33]. Nowadays, human exposure to BPS is increasing in most countries all over the world, and this trend was confirmed by finding this substance in food products and water, as well as in human blood and urine samples [11,34,35]. Moreover, previous investigations have shown that BPS has deleterious endocrine effects within the same concentration range as BPA [36]. For instance, BPS potentiates the action of oestrogens and has a negative impact on a wide range of physiological processes. In reproductive organs, BPS significantly increases the size of the uterus in female rats [37], causes reproductive dysfunctions, interferes with the maturation of oocytes, alters plasma sex hormone levels and gene transcription in the hypothalamic-pituitary-gonad axis, and depletes the activity of antioxidant enzymes [36,38]. BPS also affects the gastrointestinal tract, namely by inhibiting the activity of digestive enzymes [39], and it causes necrotic, apoptotic, oxidative and genotoxic changes in blood cells [40,41]. BPS also shows neurotoxic effects in the central nervous system, and exposure to this substance during foetal life alters behaviour later in adulthood [42,43]. Although mutagenic effects of BPS are unconfirmed, it may still cause DNA damage [44].

This study focuses on the effects of BPA and BPS in laboratory animals. The possible differences between animal studies and those transposed to humans, as well as the differences in exposure levels of these compounds, have been widely discussed in the scientific literature. There are in vivo processes that do not reflect currently known mechanisms identified in vitro. This is due to previously unknown pathways, as well as to the complexity of the in vivo cellular and system interactions.

The short-term exposure to bisphenols reported in this in vivo study may be a limitation compared to lifelong exposure to these toxins in the real life. However, our findings following acute exposure of experimental animals to bisphenols may predict that similar effects are likely to occur in humans exposed to these compounds throughout life.

Previous reports in the literature show that BPA negatively impacts GI tract function due to epithelial barrier damage and sub-epithelial inflammation, as well as by causing changes in the exocrine secretion, sensory inputs to the CNS, and peristalsis [13,14,15]. It has also been previously reported that dietary BPA uptake significantly reduces colonic microbial diversity and alters microbial structural composition. It was noted that the disruption of intestinal chemistry and physical and biological barrier functions is related to increased colonic permeability following BPA exposure [45]. There is, however, a lack of information regarding the molecular pathways and cellular implications of these changes, as well as whether BPS shares the same potency and/or mechanisms operated by BPA in the GI tract. The current study, for the first time, showed that both BPA and BPS inhibit cholinergic neurotransmission in the myenteric plexus of the colon of CD1 mice, with BPA exhibiting a 10-fold higher potency than BPS. Regarding pharmacokinetics and metabolism, BPA and BPS present some differences. BPS is absorbed more rapidly and eliminated more slowly compared to BPA. After ingestion, both compounds are metabolised in the liver. BPA is metabolised by UDP-glucuronosyltransferase 2B15 enzymes (UGTs), which BPA glucuronide metabolites are eliminated by sweat and/or urine, while BPS is mainly metabolised into BPS-glucuronide by the liver enzyme UGT1A9 with a smaller proportion being metabolised in the intestine by the UGT1A10 [46,47].

The mechanism underlying the inhibitory effects of BPA and BPS on colonic myenteric neurotransmission may stem from toxicity towards intramuscular spindle-shaped adenosine-releasing ICCs modulating ACh release at the tripartite myenteric synapse. This was suggested because in vitro application of equipotent concentrations of BPA (30 µm) and BPS (300 µm) (1) decreased the spontaneous myographic activity without affecting carbachol-induced contractions of CD1 mice colon, and (2) reduced [^3^H]ACh from stimulated myenteric neurons in parallel with deficits in extracellular adenosine accumulation, without a comparable modification in the concentration of the gliotransmitter (ATP). Prevention of BPA- and BPS-induced inhibition of [^3^H]ACh release by blocking voltage-sensitive Ca_v_3 (T-type) on ICCs with NNC-55-936 without effects being observed with the glial cell metabolic uncoupler, sodium fluoroacetate, or the FLC blocker, apamin, strengthens the theory that adenosine-releasing ICCs may be the main target of BPA and BPS in the myenteric plexus of CD1 mice colon. The confocal microscopy assessment of LM-MP preparations of the colon of CD1 mice subjected to 15-day treatment with BPA or BPS in the drinking water confirmed that hypothesis. We observed a partial loss of Ano-1-positive spindle-shaped interstitial cells of Cajal (ICCs) in the neuromuscular layer along with a reduction in VAChT-positive cholinergic neurons in the colonic myenteric plexus of both BPA- and BPS-treated mice, without measurable changes in the density of S100β-tagged enteric glial cells. In this study, we used a 50 mg/kg b.w. dosage, which is considered a non-observed adverse effect level (NOAEL) dose for BPA in mice [48,49]. As such, this means that the same dose of BPS is not free of neurotoxic effects and, thus, this compound is not a safe substitute of BPA in the chemical industry. This dose was the basis to calculate the current US-EPA reference dose (the daily dose that the EPA considers safe for humans over a lifetime) [50]. The Oral Reference Dose (RfD) is based on the assumption that there are limits to certain toxic effects, such as cell necrosis. It is expressed in mg/kg per day and represents an estimate of the daily exposure of the human population that is unlikely to pose an appreciable risk of deleterious effects over the lifetime. It should be noted that the doses used in our short-term in vivo experiments are much higher than those considered safe for humans, which the European Food Safety Authority (EFSA) set as a tolerable daily intake (TDI) of 4 μg/kg b.w. per day [51]. It is important to emphasise that the TDI is the maximum BPA dosage that is considered safe for a lifetime exposure. In relation to BPS, one very recent study based on the dose-effect curves performed from an accurate model fitting using the Benchmark Dose Software (version 3.3.2 released by the United States Environmental Protection Agency-USEPA, accessed in 1 March 2023; the software is available from https://www.epa.gov/bmds/download-bmds) determined the recommended lower limit of the benchmark dose (BMDL) for BPS, the RfD of 0.37 ng/kg-b.w. per day [52]. It is also worth noting that the circulating levels of bisphenols commonly reported in humans exceed the circulating levels extrapolated from acute exposure in laboratory animals [50].

Recently, we showed that BPA might differently affect peripheral neurons depending on the organ and tissue segments under consideration. For instance, BPA increased the number of VAChT-positive nerve fibres in porcine heart and uterus [19,53], but it decreased the amount of cholinergic neurons in the stomach and in the small intestine of pigs, as well as in the stomach of mice [16,20]. The latter findings agree with the current results focusing on LM-MP preparations of the mid colon of CD1 mice subjected to a 15-day treatment with BPA or BPS (50 mg/kg b.w. per day) in the drinking water. An increase in the proportion of VAChT-positive myenteric neurons normalised by the total amount of immunoreactive neurons labelled with the pan-neuronal marker, PGP 9.5, has been observed in transverse sections of the distal colon of mice exposed to BPA or BPS (50 mg/kg b.w.) for 3 months in the drinking water [20]. However, time-dependent analogies between apparently conflicting results must be interpreted with caution, considering differences in the experimental procedures, such as the use of distinct parts of the colon (e.g., mid vs. distal segments; isolated LM-MP vs. transverse sections), different visualisation methods (confocal vs. epifluorescence microscopy) and distinctive normalisation procedures (corrected total cryosection fluorescence vs. immunoreactivity against a pan-neuronal marker). Considering the latter, the proportion of VAChT-positive neurons normalised by staining of the pan-neuronal marker may be biased because both BPA and BPS significantly (*p* < 0.05) decreased the entire enteric neuronal population in the myenteric plexus of 3-month-treated mice [20]. In this case, the neuronal loss was also more visible with BPS than with BPA for an unidentified reason.

Similarly to the current results, Sarkar et al., (2016) [13] demonstrated that BPA (10–320 µm) decreased the duodenum motility in the rat. Using a pharmacological approach, these authors suggest that inhibition of in vitro duodenal contractions may be partially due to activation of NO-induced soluble guanylyl cyclase activation and α-adrenergic signalling pathways in smooth muscle fibres. Besides the involvement of the NO-mediated pathway [54], smooth muscle relaxation caused by bisphenols in various organs [55,56,57] may also involve inhibition of L-type Ca^2+^ channels [58] and/or interference with intracellular Ca^2+^ signalling [59]. On the other hand, BPA-induced uterine relaxation may result from downregulation of contractile proteins involving oxytocin and prostaglandin-related pathways [60]. None of these mechanisms explains the inhibitory role of BPA and BPS on electrically evoked [^3^H]ACh and adenosine release from LM-MP of mice colon (this study), considering that the current experiments were performed under non-adrenergic non-cholinergic conditions (NANC). Thus, supplementation of Tyrode’s solution with atropine (1 μm, muscarinic antagonist), prazosin (1 μm, α-adrenoceptor antagonist), propranolol (1 μm, β-adrenoceptor antagonist) and the smooth muscle relaxant, nifedipine (3 μm, L-type voltage-sensitive Ca^2+^ blocker) should abrogate any indirect mechanism operated via visceral smooth muscle fibres. Moreover, in the current study, BPA and BPS still decreased the spontaneous colonic activity, without affecting carbachol-induced myographic contractions.

While the proportion of intraganglionic nerves immunoreactive against nitric oxide synthase (nNOS) in the myenteric plexus varies with the segment of the GI tract and the inhibitory nNOS-positive fibres are relatively abundant in the jejunum followed by the stomach, duodenum and descending colon with a smaller representation in the ileum [20,61], it is undeniable that VAChT-positive cholinergic neurons are the most abundant neuronal cell population in the myenteric plexus throughout the GI tract [28]. An inverse gradient of nitrergic and purinergic inhibitory co-transmission was reported in the mouse colon [62]. While NO may be responsible for sustained relaxations required for storage (proximal colon), the purinergic response causes sharp transient relaxations required for coordinated propulsion (distal colon) [63]. Interestingly, BPA decreased nNOS-immunoreactivity in porcine enteric neurons; yet, while the duodenum is relatively spared from the neurotoxic action of BPA, this toxin slightly increased the percentage of nNOS-positive neurons in the colon [16]. These changes could indirectly contribute to BPA-inhibition of neuromuscular transmission in the stimulated mouse colon, but do not explain the decrease in the spontaneous colonic activity produced by both BPA and BPS. Moreover, we showed that the cholinergic impairment might be relatively independent of volume inhibitory neurotransmission operated by nitrergic nerves under pathological conditions [26].

Purines may compensate for deficits in nitrergic neurotransmission in the colon of humans, monkeys, and murine with a partial loss of intramuscular ICCs [64]. Here, we show for the first time that the loss of VAChT-positive cholinergic neurons in the colonic myenteric plexus was accompanied by the depletion of intramuscular spindle-shaped Ano-1-positive ICCs in CD1 mice exposed to BPA and BPS for 15 days in the drinking water. The current results show that even if an ICC deficiency is not yet established, acute in vitro incubation with BPA and BPS decreases the activity of pacemaker ICCs in the mouse colon. This was inferred because BPA and BPS significantly reduced the spontaneous myographic activity (with no effect on carbachol-induced contractions) of the mouse colon and caused a substantial reduction in the release of adenosine by these cells, without any parallelism with the ATP concentration from enteric glial cells [26]. Involvement of adenosine-releasing ICCs on colonic dysmotility caused by BPA and BPS was further confirmed in [^3^H]ACh release experiments, because the toxic effect of these xenobiotics was fully prevented by blockage of ICC activity with the voltage-sensitive Ca_v_3 (T-type) inhibitor, NNC-55-936, but not when the function of other non-neuronal cells was affected (with sodium fluoroacetate or apamin). Reduction in neuronal excitability of myenteric nerves by BPA and BPS reproduced that found in the frog sciatic nerve [65], which could explain inhibition of [^3^H]ACh release from stimulated myenteric neurons, but this mechanism would not be sensitive to the blockage of ICC activity with NNC-55-936.

A close proximity and signalling interplay exist between excitatory enteric cholinergic nerve terminals and intramuscular ICCs to control the myenteric neuromuscular transmission in the gut [66]. The involvement of endogenous purines, i.e., ATP and adenosine, on neuromuscular transmission adaptations in the gut has been extensively reported [67]. However, so far, only a few reports have addressed their role in the crosstalk between neurons and non-neuronal cells, i.e., enteric glial cells and ICSs, in the tripartite myenteric synaptic in health and pathological conditions [26]. Uncoupling between ATP overflow and deficits in extracellular adenosine accumulation in the myenteric plexus was detected in post-inflammatory ileitis [25,26]. This feature was ascribed to feed-forward inhibition of the adenosine-forming enzyme, ecto-5′-nucleotidase/CD73, and to the upregulation of adenosine deaminase subsequent to infiltration of inflammatory cells in the rat ileum [25]. Interestingly, adenosine neuromodulation deficiency paralleled the partial loss of intramuscular ICCs in the inflamed myenteric plexus [26], thus resembling the current findings in the colon of CD1 mice exposed to BPA and BPS for 15 days in the drinking water. It has been proven that endogenous adenosine preferentially activates high-affinity excitatory A_2A_ receptors on cholinergic nerve terminals [68,69,70]. Activation of pre-junctional A_2A_ receptor facilitates nerve-evoked [^3^H]ACh release and fosters a positive feedback mechanism resulting in increases in this transmitter release by a mechanism operated by muscarinic M_3_ [71] and nicotinic α3-containing [72] receptors. Therefore, it is highly likely that adenosine release deficits due to decreases in the amount and/or function of intramuscular ICCs may contribute to the cholinergic neurotransmission impairment detected after exposure to BPA or BPS. The similarity between bisphenol toxicity and the post-inflammatory condition at the cholinergic tripartite myenteric synapse is not surprising considering that both BPA and BPS exhibit marked pro-inflammatory activities [73]. It has been noted that exposure to both bisphenols modified macrophage phenotypes via stimulating the pro-inflammatory cytokines, including TNF-α, interleukin-1β and interleukin-6 [73], which were identified as factors that impair ICCs [74].

The question also arises whether the time of administration of bisphenols to mice in this experiment can be considered acute, sub-chronic or chronic exposure. At first glance, the 15-day exposure seems very short. However, one should remember that a laboratory mouse lives on average for about 3 years. Therefore, 15 days constitute 1.4% of the mouse’s total life expectancy. Assuming that the average human life expectancy is 75 years, 1.4% of the entire life is just over a year (1.05 years). Of course, such a simple conversion is an oversimplification due to the differences in general metabolism, functions of individual organs and intracorporeal metabolism of bisphenols when comparing humans with mice. However, one can assume that a 15-day period of mice exposure may be comparatively longer than sub-chronic exposure of humans up to several months. Moreover, exposure of humans to bisphenols polluting the environment usually occurs for many years. Thus, even if the Human daily intake of BPA is lower than the dose used in this experimental setting [75], one cannot rule out that the attack of bisphenols to the Human enteric nervous system shares a similar mechanism to that hypothesized in the mouse (this study). Our prediction is that longer exposure times to bisphenols may cause even greater disturbances in ENS functioning.

## 4. Materials and Methods

### 4.1. Animals and Tissue Collection

The experiments were conducted using preparations of longitudinal muscle-myenteric plexus isolated from the mid colon of adult CD1 mice of both genders weighing about 30 g (Charles River, Barcelona, Spain; Animal facility at ICBAS, Porto, Portugal). The animals were housed under standard laboratory conditions at a temperature of 21 °C and a light (7:30 a.m.–19:30 p.m.)/dark (19:30 p.m.–7:30 a.m.) cycle, with food and clean water ad libitum. In this study, the standard plastic water bottles were replaced by glass drinking bottles throughout the experimental protocol. In some experiments, the drinking water was supplemented with BPA (50 mg/kg b.w.) or BPS (50 mg/kg b.w.) for 15 days immediately before the sacrifice of the animals and tissue collection. To this end, glass water bottles were filled once daily with 24 mL of freshly prepared clean water containing 600 mg BPA or BPS. The 50 mg/kg b.w. is considered the non-observed adverse effect level (NOAEL) dose for BPA in the mouse [48,49]. All procedures received approval from the Direção Geral de Alimentação e Veterinária and the Ethics Committee and Animal Welfare Caring Organisation of ICBAS-UP (Decision No. 224/2017/ORBEA). All animal tests complied with the ARRIVE guidelines 2.0 for reporting animal experiments [76].

Immediately after the sacrifice of the animals by decapitation followed by bleeding, the mid colon was collected and placed in oxygenated (95% O_2_ and 5% CO_2_) physiological Tyrode’s solution comprising KCl 2.7 mm, CaCl_2_ 1.8 mm, NaH_2_PO_4_ 0.4 mm, MgCl_2_ 1 mm, NaCl 137 mm, NaHCO_3_ 11.9 mm, glucose 11.2 mm and choline chloride 0.001 mm. After removing the mesenteric fat, the colon was opened along the mesenteric border and pinned onto a Sylgard™ 184 Silicone-lined Petri dish (World Precision Instruments Ltd, Hertfordshire SG4 0TJ, UK), with the mucosal side facing up. The mucosal and submucosal layers were then carefully removed using tweezers to expose the muscular layer of the colon. Next, approximately 3 cm-long strips of muscle containing the longitudinal muscle layer and myenteric plexus (LM-MP) were cut from the mid-colon.

### 4.2. [^3^H]Acetylcholine Release

The methods for labelling the preparations and measuring the evoked release of [^3^H]-Acetylcholine ([^3^H]ACh were detailed in prior descriptions [26,68,77] and used with minor modifications. Briefly, isolated longitudinal muscle-myenteric plexus (LM-MP) strips were sectioned and mounted in a semi-automated 12-sample superfusion system (SF-12 Suprafusion 1000, Brandel, Gaithersburg, MD, USA) heated at 37 °C. The preparations were continuously superfused with gassed (95% O_2_ and 5% CO_2_) Tyrode’s solution. Following an equilibration period, the nerve terminals were labelled for 40 min with 1 µm of [^3^H]-choline (specific activity 2.5 µCi/nmol) under electrical field stimulation (EFS; 1 Hz frequency, 1 ms pulse width, 75 mA). After the loading phase, the tissues were washed out with Tyrode’s solution supplemented with the choline uptake inhibitor, hemicholinium-3 (10 µm). From this point onwards, Tyrode’s solution was also supplemented with atropine (1 μm, muscarinic antagonist), prazosin (1 μm, α-adrenoceptor antagonist), propranolol (1 μm, β-adrenoceptor antagonist) and the smooth muscle relaxant, nifedipine (3 μm, L-type voltage-sensitive Ca^2+^ blocker), to ensure that the experimental procedure took place under non-adrenergic non-cholinergic conditions (NANC).

Tritium outflow was measured using liquid scintillation spectrometry (TriCarb2900TR, Perkin Elmer, Boston, MA, USA; % counting efficiency: 56 ± 2%) in bath samples that were automatically collected every 1 min using the SF-12 suprafusion system. [^3^H]ACh release was induced twice by EFS (S_1_ and S_2_); each stimulation period was delivered at a 5 Hz frequency (200 square wave pulses of 1 ms duration). The area of the peak corresponding to evoked [^3^H]ACh release was determined by calculating the sum of the differences between the total radioactivity in the four samples collected after stimulation and the basal tritium outflow. Baseline values were deduced by linear regression of the radioactivity decay immediately before stimulus and after it returned to baseline [26,68,77].

Drugs, like BPA and BPS, were added 8 min before S_2_ and were present up to the end of the experiments. The change in the ratio between the evoked [^3^H]ACh release during the two stimulation periods (S_2_/S_1_) relative to that observed in control situations (in the absence of test drugs) was taken as a measure of the effect of the BPA and BPS. Positive and negative values represent facilitation and inhibition of evoked [^3^H]ACh release, respectively.

### 4.3. ATP and Adenosine Release

The methods employed to measure ATP and adenosine have been detailed in previous studies [25]. In summary, experiments were conducted using an automated perfusion system for sample collecting for given time periods, therefore improving the efficacy of HPLC (with diode array detection) and bioluminescence analysis. Stimulation evoked (3000 pulses) release of adenine nucleosides (INO + ADO) and ATP; samples were collected before and after stimulus application. Bath aliquots (50–250 μL) were immediately frozen in liquid nitrogen after collection and stored at −20 °C, as the enzymes remain stable for at least four weeks. The samples were analysed within one week of collection using HPLC with diode array detection (Finigan Thermo Fisher Scientific System LC/DAD, Thousand Oaks, CA, USA), which included an Accela Pump, an Accela Autosampler, a diode array detector, and an Accela PDA running the X-Calibur software (version 2.1.0.1139) chromatography manager. The ATP content of the same samples was assessed simultaneously using the luciferin-luciferase ATP bioluminescence assay kit HS II (Roche Applied Science, Indianapolis, IN, USA). Luminescence measurements were taken with a multi-detection microplate reader (SynergyHT, BioTek Instruments, Santa Clara, CA, USA).

### 4.4. Myographic Recordings

The LM-MP strips were mounted stretched along their longitudinal axis in perfusion chambers (15 mL) connected to isometric force transducers to measure the tension of the strips. The PowerLab collecting data device (Chart 5, v.4.2; AD Instruments, Colorado Springs, CO, USA) was used to record variations in tissue tension. The tissues were preloaded with 1.0 g of tension and allowed to equilibrate for 90 min, at 37 °C, by superfusion of Tyrode’s solution. After equilibrium, the superfusion was stopped for 15 min to evaluate spontaneous tissue contractions without the application of test drugs. The contractile responses were elicited by cumulative applications of carbachol (0.01 μm–50 μm) either in the absence or presence of test drugs.

### 4.5. Immunofluorescence Staining—Confocal Microscopy Observation

LM-MP fragments were isolated from the colon of mice that were exposed (or not) to 50 mg/kg b.w. of BPA or BPS per day in the drinking water, for 15 days. Four animals were used per experimental group: Control, BPA and BPS. After the sacrifice of the animals, the LM-MP fragments were stretched in all directions onto Petri dishes coated with Sylgard™ 184 Silicone (World Precision Instruments Ltd, Hertfordshire SG4 0TJ, UK). As described before by Vieira et al., (2017) [26], the tissues were fixed in PLP solution (16 h at 4 °C). Following fixation, the tissues were cryoprotected with a solution containing anhydrous glycerol 20% and phosphate buffer 0.1 M at 4 °C and stored at −20 °C for further processing. Once defrosted, tissue fragments were washed with phosphate saline buffer (PBS) and incubated with a blocking buffer for 2 h. After blocking and permeabilisation, samples were incubated with selected primary antibodies (see Table 1) diluted in the incubation buffer (foetal bovine serum 5%, serum albumin 1%, Triton X-100 1% in PBS), at 4 °C, for 48 h. For double immunostaining, antibodies were combined before application to tissue samples. It should be noted that immunofluorescence staining of ICCs using antibodies against Ano-1 required acetone (100%, for 10 min at −20 °C) as tissue fixative, which complicates double immunostaining with other primary antibodies. After washing out the primary antibodies with PBS supplemented with Triton X 1% (3 cycles of 10 min), the tissue samples were incubated in the dark with species-specific secondary antibodies (2 h, at room temperature). Finally, LM-MP fragments were attached to optical-quality glass slides using Fluoroshield™, as mounting media (Sigma, St Louis, MO, USA) and stored at 4 °C. A laser scanning confocal microscope (Olympus FV1000; Olympus, Tokyo, Japan) was used for observation and analysis.

### 4.6. Materials and Solutions

Atropine, bisphenol A, bisphenol S, choline chloride, paraformaldehyde (prills), lysine, sodium periodate, anhydrous glycerol, foetal bovine serum, prazosin hydrochloride (Sigma, St Louis, MO, USA); serum albumin, triton X-100, potassium dihydrogen phosphate (KH_2_PO_4_) (Merck, Darmstadt, Germany); Sodium Fluoroacetate (Supelco, Darmstadt, Germany); NNC-55-0396, nifedipine (Tocris Bioscience, Bristol, UK); Apamin was from Abcam Biochemicals (Cambridge, UK); [methyl-^3^H]-choline chloride (ethanol solution, 80 Ci mmol^−1^) (Amersham, UK); ATP bioluminescence assay kit HS II (Roche Applied Science, Indianapolis, IN, USA).

Bisphenol A, bisphenol S and prazosin hydrochloride were prepared in ethanol; nifedipine in DMSO and all the other drugs were prepared in distilled water. All stock solutions were stored as frozen aliquots at −20 °C. Dilutions of these stock solutions were made daily, and appropriate solvent controls were performed. No statistically significant differences between control experiments, made in the absence or in the presence of the solvents at the maximal concentrations used (0.5% *v*/*v*), were observed. The pH of the perfusion solution did not change with the addition of the drugs in the maximum concentrations applied to the preparations.

### 4.7. Presentation of Data and Statistical Analysis

The values are expressed as mean ± SEM, with *n* indicating the number of animals used for each of the experiments. Statistical analysis was performed using ordinary one-way ANOVA, uncorrected Fisher’s LSD, with a single pooled variance. *p* < 0.05 represents significant differences. The power analysis was a minimum of 0.8.

## 5. Conclusions

In conclusion, the data suggest that BPA and its analogue BPS inhibit cholinergic neurotransmission at the tripartite colonic myenteric synapse of CD1 mice by targeting adenosine-releasing ICC pacemaker cells. One may speculate that the mechanism underlying impairment of the myenteric neuromuscular transmission in the presence of BPA or BPS involves deficits in the adenosine A_2A_ receptor excitatory tonus, which might be critical to trigger the positive feedback loop resulting in facilitation of ACh release through the activation of muscarinic M_3_ and nicotinic α3-containing receptors on cholinergic nerve terminals. Research into the role of these previously unpredicted molecular targets may be worth pursuing in the future, given their putative implications in the prevention and/or clinical management of BPA/BPS-intoxicated individuals. Although the current study demonstrated that short-term in vitro application of BPA is 10-fold more neurotoxic than BPS, these compounds are about equipotent when given per os for 15 days to living animals, which questions the latter compound as a safer alternative for industrial processing of goods designed for human use.

## Figures and Tables

**Table 1:** The list of primary and secondary antibodies.

Primary Antibodies				
Antigen	Code	Species	Working Dilution	Supplier
S100β	Ab868	Rabbit (rb)	1:400	ABCAM
Ano-1	Ab53212	Rabbit (rb)	1:100	ABCAM
VaChT	AB1588	Guineapig (gp)	1:500	Chemicon
**Secondary antibodies**				
**Reagents**			**Working Dilution**	**Supplier**
Alexa Fluor 488, anti-rb			1:1000	Molecular probes
TRITC 568, anti-gp			1:150	Jackson Immuno Res.

The images were saved in TIFF format at the same resolution and, subsequently, analysed with the ImageJ^®^ software version 1.46r (National Institutes of Health) to quantify the density of stained cell constituents in the LM-MP. Systematic measurements were taken for settings such as area, integrated density, and mean grey value in all analysed images (background settings were obtained from an area of the section untreated with the primary antibody). The obtained values were used to calculate the corrected total cryosection fluorescence (CTCF) using a formula published on the website (https://theolb.readthedocs.io/en/latest/imaging/measuring-cell-fluorescence-using-imagej.html, accessed on 21 August 2025).

## Data Availability

The original contributions presented in this study are included in the article. Further inquiries can be directed to the corresponding authors.
